# Dual roles of SBSN in renal cancer progression and tumor thrombus: Cell-autonomous NF-κB-CD44 axis and IFI6-driven paracrine angiogenesis

**DOI:** 10.1016/j.isci.2026.116905

**Published:** 2026-07-23

**Authors:** Yuedian Ye, Jinbin Xu, Weihao Liu, Zhansen Huang, Xiaoming Li, Jiang Li, Yuanpeng Liao, Sam Un Cheong, Zifeng Xu, Gengguo Deng, Tiantian Wang, Jinming Di

**Affiliations:** 1 Department of Urology, The Third Affiliated Hospital, Sun Yat-sen University, Guangzhou, Guangdong, China; 2 Department of Medical Oncology, The Third Affiliated Hospital, Sun Yat-sen University, Guangzhou 510630, China; 3 Department of Urology, Nanfang Hospital, Southern Medical University, Guangzhou, China

**Keywords:** renal cell carcinoma, SBSN, tumor thrombus, tumor progression, angiogenesis

## Abstract

Renal cell carcinoma (RCC) is highly vascular and prone to venous invasion and tumor thrombus (TT) formation, though its underlying molecular mechanisms remain unclear. In this study, we found suprabasin (*SBSN*) was upregulated in RCC and enriched in TT, associated with poor prognosis. SBSN promoted RCC cell proliferation, migration, and invasion via the NF-κB-CD44 axis, and its knockdown inhibited tumor growth and affected epithelial-mesenchymal transition *in vivo*. Clinically, SBSN expression in RCC tissue positively correlates with microvessel density. Mechanistically, SBSN drove angiogenesis paracrinally by upregulating interferon alpha-inducible protein 6 (*IFI6*) in endothelial cells, which in turn inhibits endothelial cell apoptosis and promotes tube formation. Collectively, SBSN acts both intrinsically (NF-κB-CD44 axis) to boost tumor aggressiveness and extrinsically (SBSN-IFI6 axis) to promote angiogenesis. This study positions SBSN as a potential prognostic biomarker and therapeutic target to disrupt both tumor growth and vascular support in advanced RCC.

## Highlights

• SBSN is overexpressed in RCC and tumor thrombus and correlates with poor patient prognosis
• SBSN activates NF-κB-CD44 axis to boost RCC cell proliferation, migration, and invasion
• Tumor-secreted SBSN induces IFI6 to block endothelial apoptosis and stimulate angiogenesis
• SBSN is a dual oncogene promising for RCC prognosis and targeted therapy

## Graphical abstract

**Figure:**
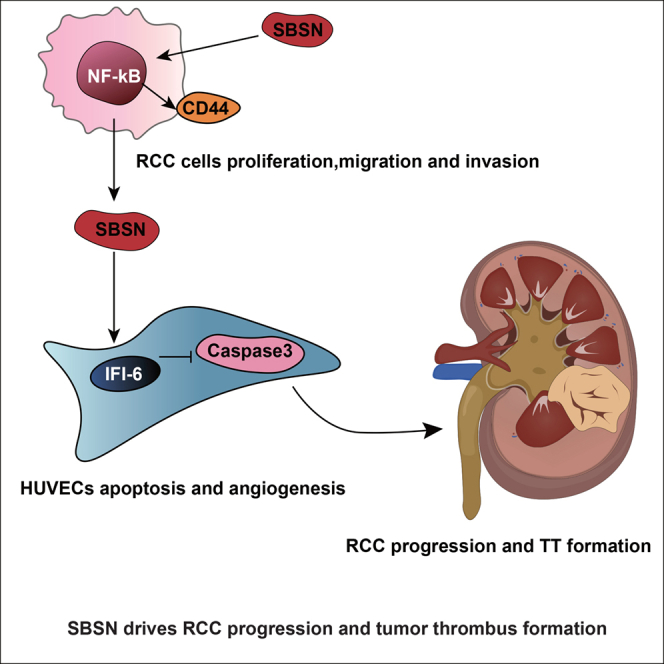

## Teaser

biological sciences; cell biology

## Introduction

Renal cell carcinoma (RCC), a common malignancy arising from the renal epithelium, accounted for an estimated 431,000 new cases and 179,000 deaths worldwide annually.^1^^,^^2^ Defined by its histologic diversity encompassing over ten subtypes, RCC is predominated by clear cell RCC (ccRCC), which constitutes roughly 75% of tumors. While surgical resection cures localized disease, approximately 30% of patients develop distant metastasis during follow-up, and nearly 10% succumb to disease progression within 5 years post-surgery.^3^ RCC is highly angiogenesis-dependent, a hallmark validated by the clinical utility of anti-angiogenic therapies.^4^ A particularly aggressive manifestation of RCC is venous tumor thrombus (TT), occurring in 4%–10% of locally advanced cases and dramatically complicating surgical management with worsening prognosis.^5^ The formation of TT exemplifies a critical yet poorly understood biological process: the intense crosstalk between tumor cells and the vascular endothelium, which is essential for understanding RCC progression and developing novel therapeutic strategies.^6^

Emerging evidence highlights the pivotal role of the nuclear factor-κB (NF-κB) signaling in RCC progression, wherein aberrant NF-κB activation not only promotes tumor cell proliferation but also orchestrates a pro-invasive microenvironment.^7^^,^^8^ It enhances tumor cell invasiveness by upregulating extracellular matrix (ECM) receptors, thereby promoting adhesion to collagen and fibronectin.^9^^,^^10^ This adhesion subsequently triggers cytoskeletal rearrangement and matrix metalloproteinase (MMP) secretion, which remodels the ECM and facilitates tumor cell infiltration into surrounding tissues.^11^^,^^12^ However, the upstream regulators that orchestrate NF-κB activity in the context of RCC invasion remain incompletely defined.

Critically, the development of TT extends beyond mechanical obstruction, representing a pathological process deeply intertwined with dysregulated angiogenesis and active vascular remodeling.^13^ The formation and stabilization of TT require the adhesion of circulating tumor cells to the vascular endothelium, which is facilitated by dynamic crosstalk mediated by secreted factors from both tumor and endothelial cells.^14^ These paracrine and juxtacrine signals promote endothelial cell survival, proliferation, and altered barrier function, thereby fostering a pro-angiogenic niche. This niche supports the expansion of the TT and provides a conduit for metastatic dissemination.^15^ Consequently, elucidating the key secreted proteins that orchestrate this tumor-vascular interface is essential for understanding the biology of RCC progression and its most aggressive local manifestation.

*SBSN*, a gene located at the q13 locus of chromosome 19 with incompletely defined biological function, was initially characterized by its expression in the basal layers of stratified epithelia, where it was proposed as a putative precursor for keratinized structure formation.^16^^,^^17^ Encoding three evolutionarily conserved secreted isoforms, SBSN acts as a signaling molecule that regulates key oncogenic pathways and drives malignant progression in diverse tumors. It’s reported that SBSN could promote bladder cancer metastasis by activating the EGFR/SRC/STAT3 pathway.^18^ Moreover, SBSN drives gastric cancer liver metastasis by activating STAT3 in tumor cells and initiating reciprocal CCL2/CCR2/JAK2 signaling with hepatic stellate cells to promote colonization.^19^ Despite these insights, research on SBSN in RCC remains scarce. In this study, we hypothesized that SBSN contributes to RCC progression by interacting with the NF-κB-CD44 signaling network. Moreover, SBSN might also influence the tumor microenvironment by modulating endothelial cell apoptosis, thus promoting angiogenesis and correlating with TT formation. Our findings not only provide new insights into the molecular orchestration of RCC progression and TT formation but also nominate SBSN as a promising prognostic biomarker and a potential therapeutic target in advanced RCC.

## Results

### SBSN is highly expressed in renal cell carcinoma and associated with poor prognosis

**Figure 1:**
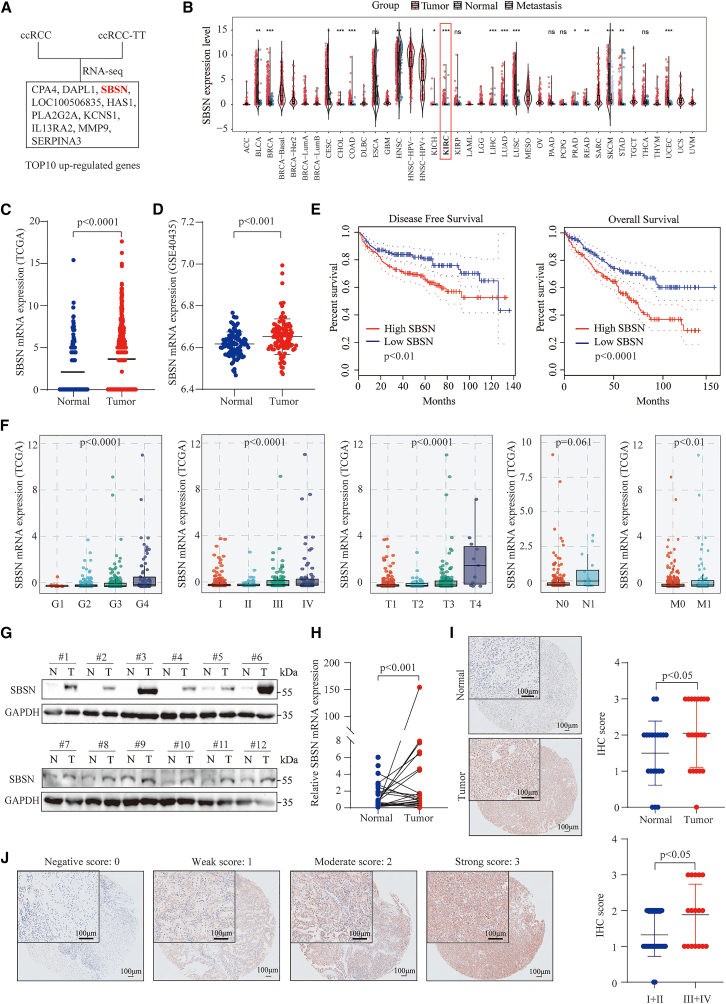
SBSN is upregulated in RCC and associated with poor prognosis (A) RNA-sequencing analysis identifying the top ten upregulated genes in ccRCC and ccRCC-TT, with *SBSN* highlighted. (B) The differential expression of *SBSN* between TCGA tumors and adjacent normal tissues was analyzed by TIMER3.0. (C and D) Scatterplots showing significantly elevated *SBSN* mRNA expression in tumor tissues compared to normal tissues in the TCGA-KIRC cohort (C) and the GSE40435 cohort (D). (E) Kaplan-Meier survival curves demonstrating that high *SBSN* expression is significantly associated with poorer disease-free survival (DFS, left) and overall survival (OS, right) in ccRCC patients. (F) Boxplots illustrating the correlation between high *SBSN* mRNA expression and advanced clinicopathological features, including tumor stage, grade, and metastasis. (G) Western blotting analysis of SBSN expression in 12 paired RCC tumor (T) and adjacent normal (N) tissues. GAPDH serves as a loading control. (H) Quantitative real-time PCR (RT-qPCR) analysis of *SBSN* mRNA expression in an independent cohort of 30 paired RCC samples. (I) Representative immunohistochemistry (IHC) images of SBSN protein expression in 20 paired adjacent normal and RCC tissues. Quantitative analysis of IHC scores confirms significantly higher SBSN expression in tumors. (J) Semi-quantitative scoring of SBSN IHC staining intensity (0–3) with example images. Correlation analysis shows that high SBSN expression (score 2–3) is more frequent in advanced-stage (III–IV, *n* = 17) RCC tumors compared to early-stage (I–II, *n* = 31) disease. All representative micrographs presented in this figure share consistent scale bars of 100 μm.

To investigate the biological function that may contribute to ccRCC-TT formation, we analyzed the expression profile from 30 primary-thrombus ccRCC pairs (NCBI Sequence Read Archive: PRJNA596338),^13^ and revealed *SBSN* as one of the top ten significantly upregulated genes in ccRCC-TT (Figure 1A). Analysis of TCGA pan-cancer data revealed elevated *SBSN* expression across multiple human cancers (Figure 1B). This suggests that *SBSN* may act as a common oncogene, contributing to tumorigenesis and progression in cancers including ccRCC (Figure 1C). This upregulation was robustly validated in another independent cohort GSE40435 (Figure 1D). Importantly, Kaplan-Meier survival analysis revealed that patients with high SBSN expression had significantly shorter disease-free survival and overall survival (Figure 1E). In addition, elevated SBSN expression correlated strongly with aggressive disease features, including advanced tumor stage, higher histological grade, and metastasis (Figure 1F). To experimentally validate the bioinformatic findings, we examined *SBSN* expression in our institutional RCC cohort. Western blotting analysis of 12 paired RCC and adjacent normal tissues revealed consistently higher SBSN protein levels in the tumors (Figure 1G). Correspondingly, RT-qPCR in an independent set of 30 paired samples demonstrated a significant increase in *SBSN* mRNA expression in RCC tissues (*p* < 0.001; Figure 1H). Immunohistochemistry further revealed more intense SBSN protein staining in tumor cells relative to normal renal epithelium (*p* < 0.05; Figure 1I). Moreover, higher SBSN expression was significantly associated with advanced tumor stage (*p* < 0.05; Figure 1J). These results collectively verify that SBSN is overexpressed at both transcript and protein levels in RCC and correlates with tumor progression.

### SBSN promotes proliferation, migration, and invasion of RCC cells *in vitro*

**Figure 2:**
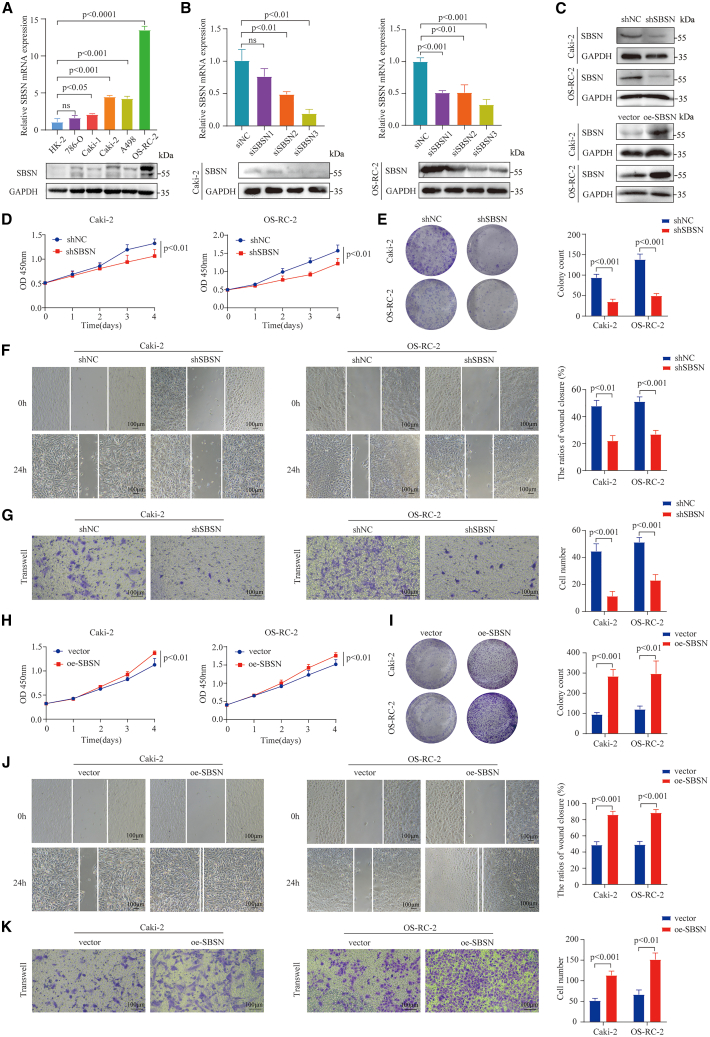
SBSN promotes the proliferation, migration, and invasion of RCC cells *in vitro* (A) Relative mRNA expression (top) and protein levels (bottom) of SBSN were analyzed by RT-qPCR and western blot (WB), respectively, in the human renal proximal tubule epithelial cell line HK-2 and a panel of RCC cell lines. (B) RT-qPCR (top) and WB (bottom) were used to validated *SBSN* knockdown efficiency in Caki-2 and OS-RC-2 cells following transfection with three independent siRNAs (siSBSN#1, siSBSN#2, siSBSN#3) compared to the scrambled negative control (siNC). (C) WB validation of SBSN overexpression (oe-SBSN) and knockdown (shSBSN). Protein levels were compared to empty vector and shNC controls, respectively, with GAPDH as the loading control. (D and E) CCK-8 and colony formation assay showed that *SBSN* knockdown significantly inhibited the proliferation of Caki-2 and OS-RC-2 cells. (F) Wound healing assay demonstrated delayed scratch closure in SBSN-knockdown RCC cells. (G) Transwell invasion assays indicated impaired invasiveness in SBSN-knockdown RCC cells. (H and I) CCK-8 and colony formation assay showed that *SBSN* overexpression significantly enhanced the proliferation of Caki-2 and OS-RC-2 cells. (J) Wound healing assay revealed accelerated cell migration of Caki-2 and OS-RC-2 cells. (K) Transwell invasion assay indicated a significant promotion of invasive capacity in SBSN-overexpressing RCC cells. Data are presented as mean ± SD of at least three independent experiments. All representative micrographs presented in this figure share consistent scale bars of 100 μm.

Initial analysis across multiple RCC cell lines revealed that *SBSN* mRNA expression was significantly elevated in most RCC-derived lines compared with the non-tumorigenic renal proximal tubule cell line HK2, with particularly high expression in Caki-2 and OS-RC-2 (Figure 2A). To investigate the functional role of SBSN in RCC progression, we constructed RCC cell lines (Caki-2 and OS-RC-2) with *SBSN* knockdown and *SBSN* overexpression. RT-qPCR and Western blotting experiments confirmed the success of *SBSN* knockdown and overexpression (Figures 2B and 2C). Functional assays demonstrated that SBSN knockdown markedly suppressed cellular proliferation. A CCK-8 assay showed that the growth rate of Caki-2 and OS-RC-2 cells was significantly reduced following SBSN knockdown (Figure 2D). In line with this, colony formation ability was also substantially impaired in SBSN-knockdown cells compared to shNC controls (Figure 2E). Further analyses revealed that SBSN regulates migratory and invasive behaviors. Wound healing assays indicated that SBSN knockdown significantly delayed scratch closure in Caki-2 and OS-RC-2 cells (Figure 2F). Invasion assays further confirmed that SBSN depletion inhibited the invasive capacities of RCC cells (Figure 2G). Conversely, SBSN overexpression promoted the proliferation (Figure 2H), clonogenicity (Figure 2I), migration (Figure 2J), and invasion (Figure 2K) in Caki-2 and OS-RC-2 cells. Collectively, these *in vitro* findings demonstrate that SBSN acts as an oncogenic driver in RCC progression.

### Transcriptomic profiling reveals that SBSN drives RCC progression through NF-κB pathway activation and subsequent CD44 upregulation

**Figure 3:**
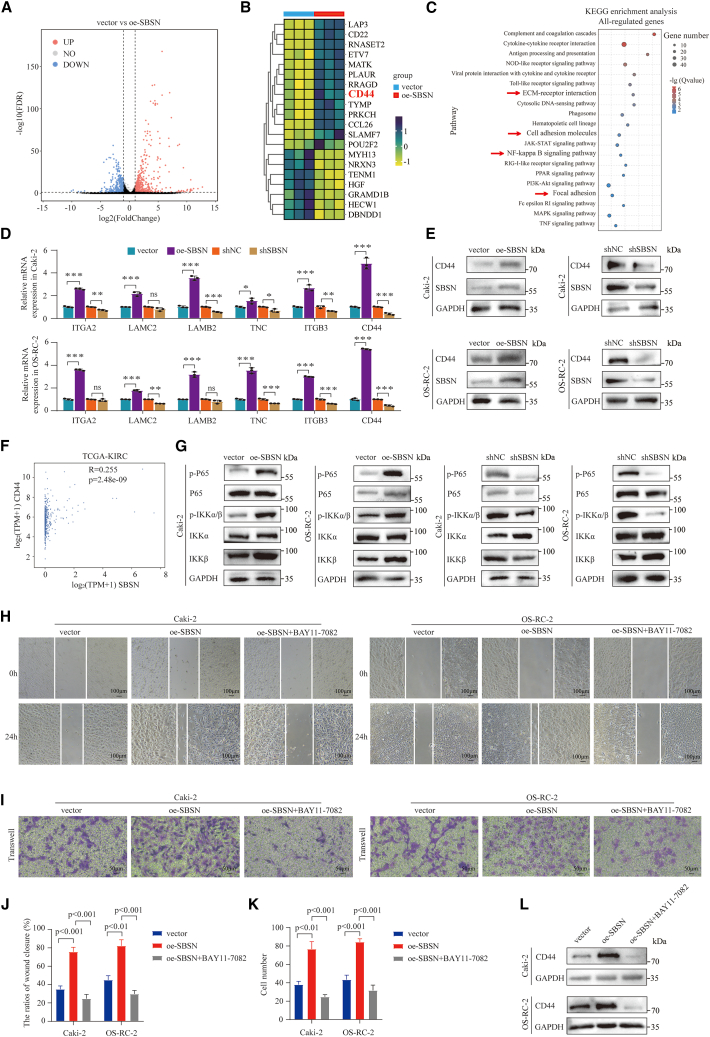
RNA sequencing analysis of SBSN-overexpressing RCC cells and validation of downstream targets (A) Volcano plot displaying DEGs in OS-RC-2 cells overexpressing *SBSN* compared to vector control. Upregulated and downregulated genes are marked in red and blue, respectively. (B) Heatmap of a subset of significantly dysregulated genes, including key candidates like *CD44*. (C) KEGG pathway enrichment analysis of all regulated genes. Bubble size represents the number of genes; color indicates the statistical significance. (D) Validation of selected DEGs involved in ECM-receptor pathway by RT-qPCR in Caki-2 and OS-RC-2 cells with *SBSN* overexpression or knockdown, confirming the regulation of *CD44* mRNA levels by *SBSN*. Data are mean ± SD; ∗*p* < 0.05, ∗∗*p* < 0.01, ∗∗∗*p* < 0.001. (E) Western blotting analysis validating SBSN regulation of CD44 protein expression in both Caki-2 and OS-RC-2 cell lines. (F) Correlation analysis between *SBSN* and *CD44* mRNA expression in the TCGA-KIRC cohort (*n* = 533). Pearson correlation coefficient (R) and *p* value are shown. (G) WB analysis of the protein levels of key NF-κB signaling pathway components in the indicated cells. (H) Wound healing assay assessing the migration ability of Caki-2 and OS-RC-2 cells. Cells were transfected with vector or oe-SBSN, with or without the NF-κB inhibitor BAY11-7082 treatment. Representative images were captured at 0 h and 24 h. (I) Transwell invasion assays evaluating the invasive capacity of Caki-2 and OS-RC-2 cells. Invaded cells were stained and visualized. (J) Quantitative analysis of the wound closure ratio from the migration assays shown in (K) Quantitative analysis of the invaded cell numbers from the transwell assays. (L) Western blotting analysis of CD44 protein levels in Caki-2 and OS-RC-2 cells. Protein lysates were harvested from cells transfected with vector or oe-SBSN, with or without treatment of the NF-κB inhibitor BAY11-7082. All representative micrographs presented in this figure share consistent scale bars of 100 μm.

To elucidate the molecular mechanisms by which SBSN drives RCC progression, we performed RNA sequencing on OS-RC-2 cells overexpressing *SBSN* (oe-SBSN). Volcano plots were generated for all differentially expressed genes (DEGs) by comparing the vector with the oe-SBSN OS-RC-2 cell and identified 926 DEGs (Figure 3A). DEGs were identified with a threshold of FDR <0.05 and |log2FoldChange| > 1. Among the upregulated genes, *CD44* gene integral to both highlighted pathways-emerged as a top candidate (Figure 3B). KEGG pathway enrichment analysis of these DEGs revealed significant involvement in cancer-related pathways, including the ECM-receptor interaction and the NF-κB signaling pathway (Figure 3C). Subsequent examination of DEGs within ECM-receptor interaction pathway specifically highlighted *ITGA2*, *LAMC2*, *LAMB2*, *TNC*, *ITGB3*, and *CD44*. RT-qPCR analysis confirmed that *CD44* was robustly upregulated upon *SBSN* overexpression in both Caki-2 and OS-RC-2 cells. Moreover, a pronounced decrease in *CD44* expression was observed following *SBSN* knockdown, reinforcing its role as a key downstream target (Figure 3D). The results were further verified by western blotting (Figure 3E), showing that CD44 was reduced in both Caki-2 and OS-RC-2 cells in response to SBSN knockdown. Consistently, analysis of the TCGA dataset demonstrated a significant positive correlation between *SBSN* and *CD44* mRNA expression in ccRCC (r = 0.255, *p* < 0.0001; Figure 3F).

To further determine whether the NF-κB signaling pathway could be activated by SBSN in RCC cells, we conducted western blotting in Caki-2 and OS-RC-2 cells. The consistent results showed that over-expression of *SBSN* promoted, while knockdown of *SBSN* attenuated, the phosphorylation levels of IKKα/β and p65. And SBSN had no significant effect on the expression of total protein of IKKα/β, and p65 (Figure 3G). To validate the functional necessity of the SBSN-NF-κB-CD44 axis, we performed rescue experiments using oe-SBSN transfection combined with BAY11-7082 (an NF-κB inhibitor) treatment. As shown in the wound healing and transwell assays, the enhanced migration and invasion capabilities induced by SBSN overexpression were significantly attenuated following treatment with BAY11-7082 (Figures 3H, 3I, 3J and 3K). Furthermore, western blotting analysis confirmed that the high CD44 protein levels in SBSN overexpression cells were effectively reduced by treatment with BAY11-7082 (Figure 3L). These data suggest that SBSN exerts its oncogenic effects, at least in part, by modulating the ECM-receptor interaction and NF-κB pathways, with CD44 as a critical downstream effector.

### SBSN expression is elevated in RCC tumor thrombus and promotes angiogenesis via IFI6-mediated inhibition of endothelial cell apoptosis

**Figure 4:**
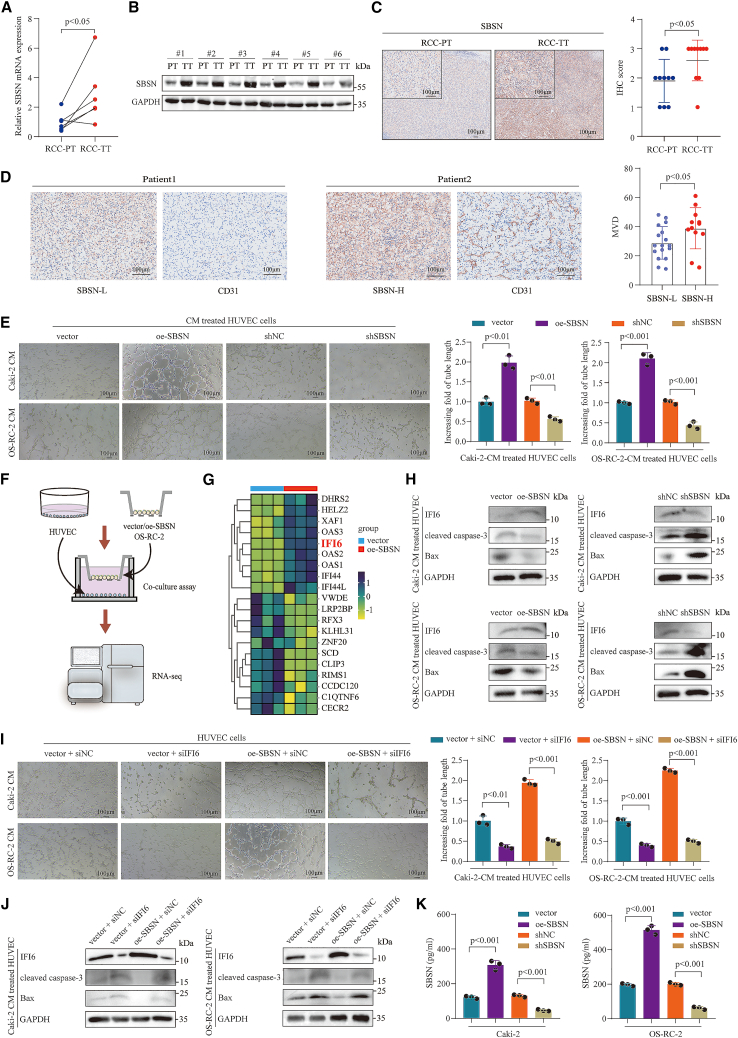
SBSN expression is elevated in RCC TT and promotes angiogenesis via IFI6-mediated inhibition of HUVEC apoptosis (A) SBSN mRNA expression levels in renal cell carcinoma tumor thrombi (RCC-TT) and matched primary tumors (RCC-PT) as determined by RT-qPCR. (B) WB analysis of SBSN protein expression in representative RCC-PT and matched RCC-TT tissues of six patients. (C) Left: Representative IHC images of SBSN expression RCC-PT and RCC-TT. Right: Quantitative analysis of IHC scores confirms significantly higher SBSN expression in RCC-TT compared to RCC-PT. (D) Comparison of CD31-positive MVD between RCC tissues with high (SBSN-H) and low (SBSN-L) SBSN expression. (E) Tube formation assay of HUVECs treated with CM from Caki-2 and OS-RC-2 cells with SBSN overexpression (oe-SBSN), knockdown (shSBSN), or their respective controls. (F) Schematic of the indirect co-culture system for RNA sequencing: HUVECs were cultured with CM from OS-RC-2 cells with oe-SBSN or their controls. (G) Heatmap showing a subset of DEGs in HUVECs treated as in (F), highlighting IFI6 as a top upregulated gene in the oe-SBSN group. (H) WB analysis of IFI6 and apoptosis-related proteins (cleaved caspase-3, Bax) in HUVECs treated with the indicated CM. SBSN overexpression in tumor cells led to increased IFI6 and decreased cleaved caspase-3 and Bax in endothelial cells, whereas SBSN knockdown had the opposite effect. (I) Representative images of tube formation assays in HUVECs cultured with CM from Caki-2 and OS-RC-2 cells. Quantification of the tube formation capacity is shown in the right. (J) Western blotting analysis of IFI6 and apoptosis-related proteins (cleaved caspase-3, and Bax) in HUVECs treated with CM from Caki-2 and OS-RC-2 cells. (K) Quantification of SBSN protein levels in the CM of Caki-2 and OS-RC-2 cells by ELISA. All representative micrographs presented in this figure share consistent scale bars of 100 μm.

Given the association of SBSN with poor prognosis and advanced disease, we investigated its potential role in vascular invasion and angiogenesis, hallmarks of aggressive RCC. Analysis of matched patient specimens revealed that SBSN expression was significantly upregulated in RCC TT compared to primary tumors (PTs), as demonstrated by both RT-qPCR (Figure 4A) and western blotting (Figure 4B). Consistently, immunohistochemical analysis of paired RCC TT and PT samples from our cohort further supported the significant upregulation of SBSN in TT (Figure 4C). Clinically, RCC with high SBSN expression (SBSN-H) exhibited greater microvessel density (MVD) than those with low SBSN expression (SBSN-L) (Figure 4D). The association prompted us to test the functional role of SBSN in angiogenesis *in vitro*. Conditioned medium (CM) from SBSN-overexpressing RCC cells significantly enhanced the tube-forming ability of human umbilical vein endothelial cells (HUVECs), whereas medium from SBSN-knockdown cells impaired it (Figure 4E). To identify the mechanism mediating this pro-angiogenic effect, we performed RNA sequencing on HUVECs treated with conditioned media from vector and SBSN-overexpressing OS-RC-2 cells (Figure 4F). This analysis identified *IFI6* as a key gene upregulated in HUVECs by tumor-derived SBSN (Figure 4G). Western blot analysis confirmed that SBSN-overexpressing tumor cell medium increased IFI6 protein levels in HUVECs, while simultaneously decreasing the levels of pro-apoptotic proteins such as cleaved caspase-3 and Bax. To further substantiate the role of IFI6 in regulating angiogenesis, we knocked down *IFI6* in HUVECs, and then treated them with the CM that collected from oe-SBSN RCC cells to assess the subsequent effects. The tube formation assay results demonstrated that reducing IFI6 levels in HUVECs significantly inhibited the angiogenic capability induced by the SBSN-overexpressing medium (Figure 4I). Furthermore, western blotting analysis revealed that IFI6 knockdown in HUVECs increased the protein levels of apoptosis markers. (Figure 4J). Also, ELISA assay was used to quantify SBSN protein levels in CM from vector control and SBSN-overexpressing Caki-2 and OS-RC-2 cells. SBSN levels were significantly elevated in CM from oe-SBSN RCC cells (Figure 4K). These findings indicate that tumor-derived SBSN promotes angiogenesis by upregulating the anti-apoptotic factor IFI6 in endothelial cells, thereby enhancing their survival and tube-forming capacity.

### SBSN accelerates the progression of RCC *in vivo*

**Figure 5:**
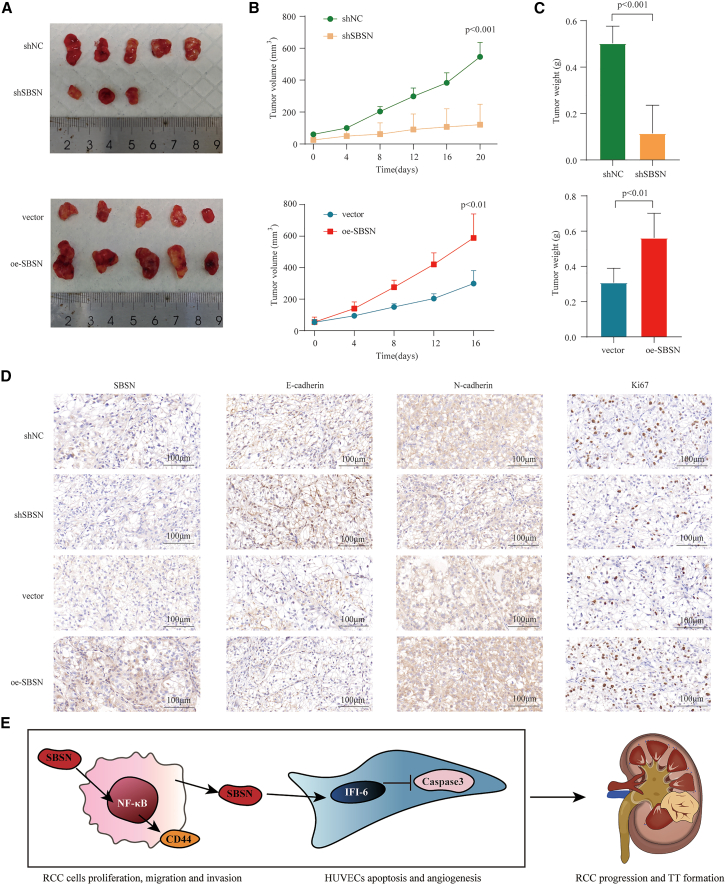
SBSN promotes *in vivo* tumor growth and RCC progression via cell-autonomous and paracrine mechanisms (A) Representative images of subcutaneous tumors in shNC/shSBSN (upper) and vector/oe-SBSN (lower) groups. (B) Tumor volume growth curves. (C) Tumor weight at harvest. (D) IHC staining of SBSN in xenograft tissues, confirming knockdown/overexpression efficiency. IHC staining of phenotypic markers (E-cadherin, N-cadherin, Ki67) in xenograft tissues. (E) Schematic of the mechanism of this study. All representative micrographs presented in this figure share consistent scale bars of 100 μm.

To verify the *in vivo* oncogenic role of SBSN, we established subcutaneous xenograft models using OS-RC-2 cells with *SBSN* knockdown (shSBSN) or overexpression (oe-SBSN), paired with their respective controls (shNC, vector). Visual inspection of excised tumors showed that SBSN knockdown markedly reduced tumor size (Figure 5A, top), while SBSN overexpression significantly enlarged tumors (Figure 5A, bottom). Corresponding tumor volume curves confirmed slower growth in the shSBSN group (vs. shNC; *p* < 0.001) and accelerated expansion in the oe-SBSN group (vs. vector; *p* < 0.01) (Figure 5B). Consistently, tumor weight was decreased in shSBSN tumors (vs. shNC; *p* < 0.001) and increased in oe-SBSN tumors (vs. vector; *p* < 0.01) (Figure 5C). Immunohistochemical staining validated SBSN manipulation efficiency: SBSN expression was diminished in shSBSN tissues and elevated in oe-SBSN tissues (Figure 5D). Further analysis of phenotypic markers revealed that SBSN overexpression downregulated the epithelial marker E-cadherin, upregulated the mesenchymal marker N-cadherin, and increased the proliferation marker Ki67; SBSN knockdown showed the opposite trends (Figure 5D). The schematic in Figure 5E summarizes SBSN’s dual mechanisms: in RCC cells, SBSN activates NF-κB-CD44 signaling to promote proliferation/migration/invasion; via paracrine signaling, SBSN upregulates IFI6 in HUVECs to inhibit caspase-3-induced apoptosis and enhance angiogenesis, collectively driving RCC progression and TT formation. The schematic summarizes the dual mechanisms of SBSN. In RCC cells, SBSN activates the NF-κB-CD44 signaling axis to promote proliferation, migration, and invasion. In parallel, via paracrine signaling, SBSN upregulates IFI6 in endothelial cells, thereby inhibiting caspase-3-induced apoptosis and enhancing angiogenesis. Together, these mechanisms drive RCC progression and correlate with TT formation. (Figure 5D).

## Discussion

Renal cell carcinoma is a highly vascularized malignancy characterized by a distinctive dependence on angiogenesis, which is clinically validated by the efficacy of anti-angiogenic agents as first-line therapeutics.^20^^,^^21^^,^^22^ Furthermore, RCC exhibits a remarkable propensity for venous invasion and the formation of TT, a direct manifestation of aggressive tumor-vessel interaction.^23^^,^^24^ This unique pathological feature positions RCC as an exceptional model to decipher the molecular drivers of intravasation and metastasis. Based on this rationale, our study aimed to identify key mediators of this aggressive phenotype by profiling the transcriptional differences between RCC TT and PTs. Our study identifies *SBSN* as a novel oncogenic mediator in RCC, with consistent overexpression in tumor tissues relative to adjacent normal tissues and association with unfavorable patient outcomes.

Emerging evidence identifies *SBSN* as a critical regulator of oncogenic processes.^25^^,^^26^^,^^27^ However, the specific mechanism underlying the action of SBSN in RCC has remained elusive. A core functional observation is that SBSN could promote RCC cell proliferation, migration, and invasion. RNA-sequencing analysis pinpointed enrichment of the NF-κB signaling pathway in SBSN-overexpressing cells. Similarly, it is reported that SBSN-containing extracellular vesicles drive glioma tumorigenesis by activating the NF-κB pathway through an interaction with annexin A1.^28^ A critical downstream effector of NF-κB signaling pathway is *CD44*, a transmembrane glycoprotein that serves as a principal receptor for ECM components, particularly hyaluronic acid. *CD44* upregulation has been consistently correlated with advanced tumor stage, metastasis, and poor prognosis in RCC patients.^29^^,^^30^ RNA-sequencing results demonstrated an enrichment of the ECM receptor interaction in SBSN-overexpressing cells, and correlation analysis indicated that *SBSN* showed the strongest association with *CD44* among all ECM receptor components. Mechanistically, we uncovered a novel SBSN-NF-κB-CD44 axis in RCC.

A clinically significant finding is the association of SBSN with TT formation, a complication tightly linked to dysregulated angiogenesis.^31^^,^^32^^,^^33^ We observed significantly elevated SBSN expression in TT tissues compared with primary RCC, and SBSN protein levels positively correlated with MVD within RCC tissue. These clinical observations support a pro-angiogenic role for SBSN and suggest that high SBSN expression may foster a vascular microenvironment that facilitates thrombus sustenance and progression, as well as subsequent metastatic dissemination. Mechanistically, we delineated a paracrine signaling axis whereby tumor-derived SBSN promotes endothelial survival and tubulogenesis, with *IFI6* identified as a critical downstream effector. As a well-characterized mitochondrial anti-apoptotic protein triggered by cellular stress and interferon signaling, IFI6 can be hijacked by tumor-secreted SBSN to strengthen endothelial survival.^34^^,^^35^ Reduced levels of cleaved caspase-3 and Bax in HUVECs cultured with SBSN-overexpressing CM further verify that SBSN-mediated pro-angiogenic effects are partially dependent on endothelial apoptosis suppression. This represents a sophisticated tumor-stroma crosstalk, in which cancer cells secrete SBSN to augment endothelial robustness and construct a stable vascular network supporting tumor invasion and TT progression. Collectively, our findings indicate that targeting SBSN may disrupt the vascular microenvironment for TT maintenance and restrict progression in advanced RCC, with further validation in qualified preclinical models still warranted.

Our findings suggest that SBSN may represent a promising therapeutic target in advanced RCC, given its dual roles in promoting both tumor cell aggressiveness and angiogenesis. SBSN-IFI6 cascade is hypothesized to weaken the therapeutic efficacy of anti-angiogenic tyrosine kinase inhibitors (TKIs), since TKI treatment suppresses VEGF/VEGFR signaling and induces endothelial apoptosis.^36^^,^^37^ Therefore, SBSN upregulation may serve as a potential mechanism underlying TKI resistance. The identification of SBSN as a secreted factor that acts in a paracrine manner to stabilize the vascular endothelium raises the possibility of developing therapeutic strategies aimed at neutralizing extracellular SBSN. Antibody-based neutralization or peptide inhibitors targeting SBSN could potentially disrupt the tumor-vascular interface, thereby inhibiting both PT progression and the formation or stabilization of TT. However, it remains to be further verified.

In summary, our study identifies *SBSN* as a key oncogenic factor in RCC that is associated with aggressive tumor progression and TT formation. We demonstrate that SBSN enhances the malignant potential of RCC cells through the NF-κB-CD44 axis, while also driving angiogenesis via a paracrine mechanism. Specifically, tumor-derived SBSN upregulates the anti-apoptotic factor IFI6 in endothelial cells, thereby enhancing endothelial survival and tube formation. These findings elucidate a novel SBSN-IFI6 axis that contributes to vascular remodeling and supports the aggressive phenotype within the TT microenvironment. Collectively, our work establishes SBSN as a marker of aggressive disease and a promising prognostic biomarker, as well as a potential therapeutic target whose inhibition may disrupt both intrinsic tumor growth and extrinsic vascular support in advanced RCC.

### Limitations of the study

We acknowledge several limitations of this study. First of all, our *in vivo* experiments used subcutaneous xenografts, which do not fully recapitulate the renal microenvironment or TT formation; the definitive role of SBSN in driving RCC progression *in vivo* requires validation using orthotopic or patient-derived xenograft models with manipulated SBSN expression. Secondly, while we observed correlations between SBSN and IFI6, the specific receptor for SBSN on endothelial cells remains unidentified, representing a crucial gap in understanding the initial signal transduction event. Finally, our clinical correlations, while suggestive, are based on a retrospective cohort; prospective validation in larger, multi-center studies is necessary to confirm the prognostic utility of SBSN.

## Resource availability

### Lead contact

Requests for further information and resources should be directed to and will be fulfilled by the lead contact, Jinming Di (dijinm@mail.sysu.edu.cn).

### Materials availability

This study did not generate new unique reagents.

### Data and code availability

• This study analyzes existing, publicly available data, accessible at GEO and GSA-Human: Archive: GSE40435 and PRJNA596338.
• All codes were used in this study in alignment with recommendations made by authors of R packages in their respective user’s guide, which can be accessed at https://bioconductor.org.
• Any additional information required to reanalyze the data reported in this study is available from the GEO: GSE327867.

## Acknowledgments

This article was supported by the 10.13039/501100001809National Natural Science Foundation of China (no. 82272840, no. 81772752), 10.13039/501100003453Guangdong Natural Science Foundation (2021A1515010129, 2023A1515012925).

## Author contributions

Conceptualization, Y.Y., J.X., and W.L.; methodology, Z.H., X.L., J.L., and Y.L.; investigation, S.U.C. and Z.X.; writing – original draft, Y.Y.; writing – review and editing, G.D., T.W., and J.D.; funding acquisition, J.D.; supervision, J.D. All authors interpreted the results and read and approved the final version of the manuscript.

## Declaration of interests

The authors declare no competing interests.

## Declaration of generative AI and AI-assisted technologies in the writing process

No generative AI or AI-assisted technologies were used in the writing or preparation of this manuscript.

## STAR★Methods

### Key resources table

**Table:** 

REAGENT or RESOURCE	SOURCE	IDENTIFIER
**Antibodies**		

Anti-SBSN	Abcam	ab232771; RRID: AB_3712950
Anti-NF-κB p65	Cell Signaling Technology	#8242; RRID: AB_10859369
Anti-p-NF-κB p65	Cell Signaling Technology	#3033; RRID: AB_331284
Anti-cleaved caspase-3	Cell Signaling Technology	#9661; RRID: AB_2341188
Anti-IFI6	Cell Signaling Technology	#54355; RRID: AB_3712897
Anti-Bax	Cell Signaling Technology	#2772; RRID:AB_10695870
Anti-CD44	Cell Signaling Technology	#3578; RRID: AB_2076463
Anti-*p*-IKKα/β (Ser176/180)	Cell Signaling Technology	#2697; RRID: AB_2079382
Anti-IKKα	Cell Signaling Technology	#11930; RRID: AB_2687618
Anti-IKKβ	Cell Signaling Technology	#8943; RRID: AB_11024092
Anti-GAPDH	Cell Signaling Technology	#2118; RRID: AB_561053
Anti-Ki-67	Cell Signaling Technology	#12075;RRID:AB_2728830
Anti-CD31	Cell Signaling Technology	#95462; RRID:AB_3739873
HRP-conjugated secondary antibodies	Cell Signaling Technology	#7074, #7076; RRID: AB_330924
Biotinylated secondary antibodies	Zsgb-bio	#ZF-0317, ZB-2306; RRID: AB_2868454

**Bacterial and virus strains**		

Lentiviral vectors (SBSN overexpression, shRNA-SBSN, shNC, empty vector)	Shybio	# MBDZT

**Biological samples**		

RCC tissues and tumor-adjacent normal tissues	Third Affiliated Hospital of Sun Yat-sen University & Nanfang Hospital	Approval ID: II2025-450-01 (SYSU); NFEC-2022-299 (Nanfang Hospital)
RCC tumor thrombus (TT) and matched primary tumor (PT) samples	Third Affiliated Hospital of Sun Yat-sen University	Approval ID: II2025-450-01 (SYSU)

**Chemicals, peptides, and recombinant proteins**		

Polybrene	Glpbio	#28728-55-4
Puromycin	TargetMol	#53-79-2
Matrigel	Corning	#356231
Fetal bovine serum (FBS)	Gibco	#10099141C
Penicillin-streptomycin	Solarbio	#P1400
RIPA lysis buffer	Cell Signaling Technology	#9806S
Protease and phosphatase inhibitors	Roche	# PPC1010
3,3ʹ-diaminobenzidine (DAB)	Yeasen	#36201ES03

**Critical commercial assays**		

BCA assay kit	Thermo Fisher Scientific	#23227
All-in-One First Strand cDNA Synthesis Kit	Sevenbio (Beijing, China)	#SM134
2× SYBR Green qPCR MasterMix	Sevenbio (Beijing, China)	#SM143
TRIzol reagent	Invitrogen	# 15596018CN

**Deposited data**		

ccRCC-TT expression profile data	GSA Human Sequence Read Archive	PRJNA596338
ccRCC gene expression data	GEO	GEO: GSE40435, GSE327867
TCGA pan-cancer/ccRCC (KIRC) data	TCGA	–

**Experimental models: Cell lines**		

HK-2 (non-tumorigenic renal proximal tubule cell line)	ProCell Life Science and Technology Company (Wuhan, China)	# CL-0109
Caki-1 (RCC cell line)	ProCell Life Science and Technology Company (Wuhan, China)	# CL-0052
A498 (RCC cell line)	ProCell Life Science and Technology Company (Wuhan, China)	# CL-0254
OS-RC-2 (RCC cell line)	ProCell Life Science and Technology Company (Wuhan, China)	# CL-0177
786-O (RCC cell line)	ProCell Life Science and Technology Company (Wuhan, China)	# CL-0010
Caki-2 (RCC cell line)	ProCell Life Science and Technology Company (Wuhan, China)	# CL-0326
HUVECs (human umbilical vein endothelial cells)	ProCell Life Science and Technology Company (Wuhan, China)	# CP-H082Y

**Experimental models: Organisms/strains**		

BALB/c nude mice (female, 4–5 weeks old, 18-20g)	Sun Yat-sen University animal center	Approval ID: SYSU-IACUC-2025-003052

**Oligonucleotides**		

shRNA-SBSN (three independent siRNAs: siSBSN#1, siSBSN#2, siSBSN#3)	HanYi Biosciences Inc	–
Nontargeting control shRNA (shNC)	HanYi Biosciences Inc	–
qRT-PCR primers for SBSN, CD44, ITGA2, LAMC2, LAMB2, TNC, ITGB3, IFI6, GAPDH	Tsingke Biotechnology Co., Ltd	–

**Software and algorithms**		

GraphPad Prism	GraphPad Software	Version 9.0
SPSS	IBM	Version 26.0
StepOnePlus Real-Time PCR System Software	Applied Biosystems	Version 7.0
R packages	Bioconductor	https://bioconductor.org
Odyssey Infrared Imaging System Software	Bio-Rad	Version 6.1

**Other**		

RPMI 1640 medium	Gibco	#72400047
6-well transwell insert (0.4 μm polyester membrane)	Corning	#3450

### Experimental model and study participant details

#### Cells and cell culture

The non-tumorigenic renal proximal tubule cell line HK-2 and renal cell carcinoma cell lines Caki-1, A498, OS-RC-2, 786-O, Caki-2 and HUVECs were purchased from ProCell Life Science and Technology Company (Wuhan, China). All human cell lines were STR-authenticated upon arrival, after lentiviral stable cell generation, and every 20 passages per ANSI/ATCC ASN-0002 standards. All lines matched reference profiles from ATCC/DSMZ databases with ≥90% identity. Cells were screened biweekly for mycoplasma via Sigma LookOut PCR kits; no mycoplasma contamination was detected in experimental batches.

Cells were cultured in RPMI 1640 medium (Gibco) supplemented with 10% fetal bovine serum (FBS, Gibco) and 100 U/mL penicillin-streptomycin (Solarbio). Cultures were maintained in a humidified incubator at 37°C with 5% CO_2_. For stable cell line construction, Caki-2 and OS-RC-2 cells were transduced with lentiviral vectors expressing *SBSN* (SBSN overexpression), shRNA-*SBSN* (shSBSN), non-targeting control shRNA (shNC), or empty vector (vec) using polybrene (8 μg/mL). Stable clones were selected with puromycin (2 μg/mL) for 10 days and verified by Western blotting and quantitative real-time PCR.

#### Human tissue specimens and study participants

RCC tissues and corresponding tumor-adjacent normal tissues were collected from surgically excised renal specimens at Nanfang Hospital and the Third Affiliated Hospital of Sun Yat-sen University (Guangzhou, China). All participants were ethnic Han Chinese. Patients who received preoperative chemotherapy, targeted therapy, or radiotherapy prior to sample collection were excluded from this study. All patients provided written informed consent before sample collection. Four sets of patient tissue specimens were used: 12 paired fresh RCC tumor/adjacent normal tissues for SBSN western blot; 30 independent paired fresh tumor-normal tissues for SBSN RT-qPCR quantification; 6 matched primary tumor–tumor thrombus fresh pairs for western blot; 6 matched FFPE RCC tissues (paired normal-tumor and primary tumor-thrombus sections) for SBSN IHC and clinical correlation analysis. All eligible consecutive surgical patients were retrospectively enrolled without randomization. Clinical records of enrolled patients were reviewed to extract age, sex, tumor grade, metastatic status and thrombus status. Associations between patient sex and SBSN expression, as well as clinical outcomes, were analyzed in the survival and clinicopathological correlation analyses. No significant sex-based difference in SBSN expression level or prognostic impact was observed in our retrospective cohort. Detailed sex distribution of each cohort is summarized in Table S1. The study protocol was approved by the Medical Ethics Committee of the Third Affiliated Hospital of Sun Yat-sen University (Approval ID: II2025-450-01) and the Ethics Committee of Nanfang Hospital (Approval ID: NFEC-2022-299).

#### Animals

Female BALB/c nude mice (4–5 weeks, 18–20 g) were housed in SPF facilities with 12 h light/dark cycles, 22 ± 2°C temperature and 50% ± 10% humidity. Sterile feed and water were supplied *ad libitum*. Female mice were used exclusively to exclude androgen-related fighting and growth bias; sex-specific effects in males represent a study limitation (see Sex and Gender Limitation Statement). All animal experiments complied with the ARRIVE guidelines and were approved by the Institutional Animal Care and Use Committee of Sun Yat-sen University (Approval ID: SYSU-IACUC-2025-003052).^38^

#### Renal cell carcinoma subcutaneous xenograft model in nude mice

Mice were randomly divided into four groups (*n* = 5 per group): vector, oe-SBSN, shNC, and shSBSN. For subcutaneous tumor formation, 1×10^6^ stably transfected OS-RC-2 cells were suspended in 100 μL PBS and injected into the right axilla of each mouse. Tumor volume was measured every 4 days using a vernier caliper, calculated as volume = (length × width^2^)/2. After 2–3 weeks, mice were anesthetized with 0.6% pentobarbital sodium (60 mg/kg, intraperitoneal injection) and euthanized by cervical dislocation. Tumors were harvested then were weighed, and portions were fixed in formalin for IHC staining.

#### Sex and gender limitation statement

Only female nude mice were used for xenograft assays, so sex-specific tumor phenotypes in male mice remain untested and warrant future orthotopic studies with mixed-sex animals. Our retrospective Han Chinese RCC cohort included both sexes; subgroup analysis showed no significant sex-linked differences in SBSN levels, tumor thrombus or survival, yet balanced large prospective cohorts are needed for validation. Immortalized RCC and HUVEC lines lack native sex hormone signaling, thus *in vitro* SBSN pathways may not fully mirror sex-dependent tumor microenvironment interactions in patients.

### Method details

#### Western blotting assay

Total cellular or tissue proteins were extracted using RIPA lysis buffer (Cell Signaling Technology) supplemented with protease and phosphatase inhibitors (Roche). Protein concentration was quantified by BCA assay (Thermo Fisher Scientific). Equal amounts of protein (30 μg) were separated by 10% SDS-PAGE and transferred onto nitrocellulose membranes (Millipore). Membranes were blocked with 5% bovine serum albumin (BSA) in Tris-buffered saline with 0.1% Tween 20 (TBST) for 1 h at room temperature, then incubated with primary antibodies overnight at 4°C. Primary antibodies included anti-SBSN (ab232771) (Abcam), anti-NF-κB p65 (#8242), anti-p-NF-κB p65 (#3033), cleaved caspase-3 (#9661), IFI6 (#54355), Bax (#2772), CD44 (#3578), *p*-IKKα/β (Ser176/180) (#2697), IKKα (#11930), IKKβ (#8943) and GAPDH (#2118) (Cell Signaling Technology). After washing with TBST, membranes were incubated with horseradish peroxidase (HRP)-conjugated secondary antibodies (Cell Signaling Technology, #7074, #7076) for 1 h at room temperature. Protein bands were visualized using an Odyssey Infrared Imaging System (Bio-Rad).

#### Quantitative real-time PCR (RT-qPCR)

Total RNA was extracted from cells and tissues using TRIzol reagent (Invitrogen) following the manufacturer’s protocol. Complementary DNA (cDNA) was synthesized with the All-in-One First Strand cDNA Synthesis Kit (Sevenbio, Beijing, China). qRT-PCR was performed on a StepOnePlus Real-Time PCR System (Applied Biosystems) using 2× SYBR Green qPCR MasterMix (Sevenbio). The reaction conditions were: 95°C for 10 min, followed by 40 cycles of 95°C for 15 s and 60°C for 1 min. Relative gene expression levels were calculated using the 2^−ΔΔCt^ method, with GAPDH as the internal reference gene.

#### Immunohistochemical staining

Paraffin sections were deparaffinized, rehydrated, and subjected to antigen retrieval in EDTA buffer (pH 8.0) by microwave heating. Endogenous peroxidase activity was blocked with 3% H_2_O_2_ for 10 min. Sections were blocked with 5% BSA for 1 h at room temperature, then incubated with primary antibodies overnight at 4°C. Primary antibodies include SBSN, Ki-67 (#12075, Cell Signaling Technology), and CD31 (#95462, Cell Signaling Technology). After washing, sections were incubated with biotinylated secondary antibodies (ZSGB-BIO) for 30 min, followed by streptavidin-horseradish peroxidase complex (ZSGB-BIO) for 20 min. Staining was visualized with 3,3ʹ-diaminobenzidine (DAB, ZSGB-BIO), and sections were counterstained with hematoxylin. IHC scores were calculated as the product of staining intensity (0 = negative, 1 = weak, 2 = moderate, 3 = strong).

#### Cell co-culture assay

For the co-culture experiment, HUVECs were seeded in the basolateral compartment of a 6-well transwell system (0.4 μm polyester membrane, #3450, Corning). RCC cells were plated on top of the inserts. After 48 h of co-culture, HUVECs were harvested for subsequent experiments.

#### Invasion assay

The upper chamber was pre-coated with 50 μL matrigel (1:7 dilution in PBS, Corning) and polymerized at 37°C for 1 h. 1×10^4^ OS-RC-2 or Caki-2 cells were seeded in the upper chamber of Transwell inserts (8 μm pore size, Corning) with serum-free medium. The lower chamber was filled with medium containing 20% FBS. After 48 h of incubation, cells on the upper surface of the membrane were removed with cotton swabs. Migrated cells on the lower surface were fixed with 4% paraformaldehyde, stained with hematoxylin, and counted under a light microscope.

#### Wound-healing assay

Cells were seeded in 24-well plates and cultured to 90% confluence. A straight wound was created in the cell monolayer using a sterile 200 μL pipette tip. Cells were washed with PBS to remove debris and cultured in serum-free medium. Images were captured at 0 and 24 h using an inverted microscope (Olympus). The wound healing rate was calculated as (initial wound width - remaining wound width)/initial wound width × 100%.

#### RNA sequencing (RNA-seq)

RNA sequencing was performed on OS-RC-2 cells with SBSN overexpression versus empty vector control, and on HUVEC cells treated with conditioned media from these respective OS-RC-2 groups. Following total RNA extraction and quality control, libraries were sequenced on an Illumina platform. Reads were aligned to the hg38 genome using STAR, and gene counts were quantified with HT-seq against the RefSeq annotation. Differential expression was analyzed with DEGs, applying a significance threshold of FDR <0.05 and |log2FoldChange| > 1. Enriched KEGG pathways were identified from the ranked gene list using GSEA.

#### Statistical analysis

All data are presented as mean ± Standard Error of the Mean (SEM) from at least three independent experiments. Statistical analyses were performed using GraphPad Prism 9.0 (GraphPad Software) and SPSS 26.0 (IBM). Differences between the two groups were analyzed by a two-tailed Student’s *t* test. Multiple group comparisons were performed using two-way analysis of variance (ANOVA). Correlations between variables were assessed by Pearson’s correlation coefficient. Survival curves were analyzed by the Kaplan-Meier method with the log rank test.

### Quantification and statistical analysis

All statistical details are described in the figure legends and in the STAR Methods.

## Supplemental information

Document S1. Table S1

Data S1. Raw unprocessed experimental imaging files sorted into three separate subfolders, covering Figure 1G GAPDH western blot raw images, Figure 2F 0 h scratch assay replicates of OSRC2 shNC cells, and Figure 3H 0 h scratch assay replicates of OSRC2 oeSBSN overexpression cells
