# Supplementary material for: Dual roles of SBSN in renal cancer progression and tumor thrombus: Cell-autonomous NF-κB-CD44 axis and IFI6-driven paracrine angiogenesis
Source: iScience. 2026 Jul 23;29(8):116905. doi: 10.1016/j.isci.2026.116905 (PMC13427577; doi:10.1016/j.isci.2026.116905)
Supplement: Document S1. Table S1 [file mmc1.pdf]

iScience, Volume *29*

Supplemental information

Dual roles of SBSN in renal cancer progression and tumor thrombus: Cell-autonomous NF-κB-CD44 axis and IFI6-driven paracrine angiogenesis

Yuedian Ye, Jinbin Xu, Weihao Liu, Zhansen Huang, Xiaoming Li, Jiang Li, Yuanpeng Liao, Sam Un Cheong, Zifeng Xu, Gengguo Deng, Tiantian Wang, and Jinming Di

Table S1: Demographic characteristics and sex distribution of all enrolled ccRCC patient cohorts

Cohort No.	Experimental Application	Total paired cases	Male (n)	Female (n)
1	SBSN protein Western blot (tumor-normal pairs)	12	9	3
2	SBSN mRNA RT-qPCR (tumor-normal pairs)	30	21	9
3	Western blot for matched primary tumor and tumor thrombus	6	5	1
4	FFPE IHC staining and clinical prognostic analysis	29	17	12
