# Supplementary figures and images for: Dual roles of SBSN in renal cancer progression and tumor thrombus: Cell-autonomous NF-κB-CD44 axis and IFI6-driven paracrine angiogenesis
Source: iScience. 2026 Jul 23;29(8):116905. doi: 10.1016/j.isci.2026.116905 (PMC13427577; doi:10.1016/j.isci.2026.116905)

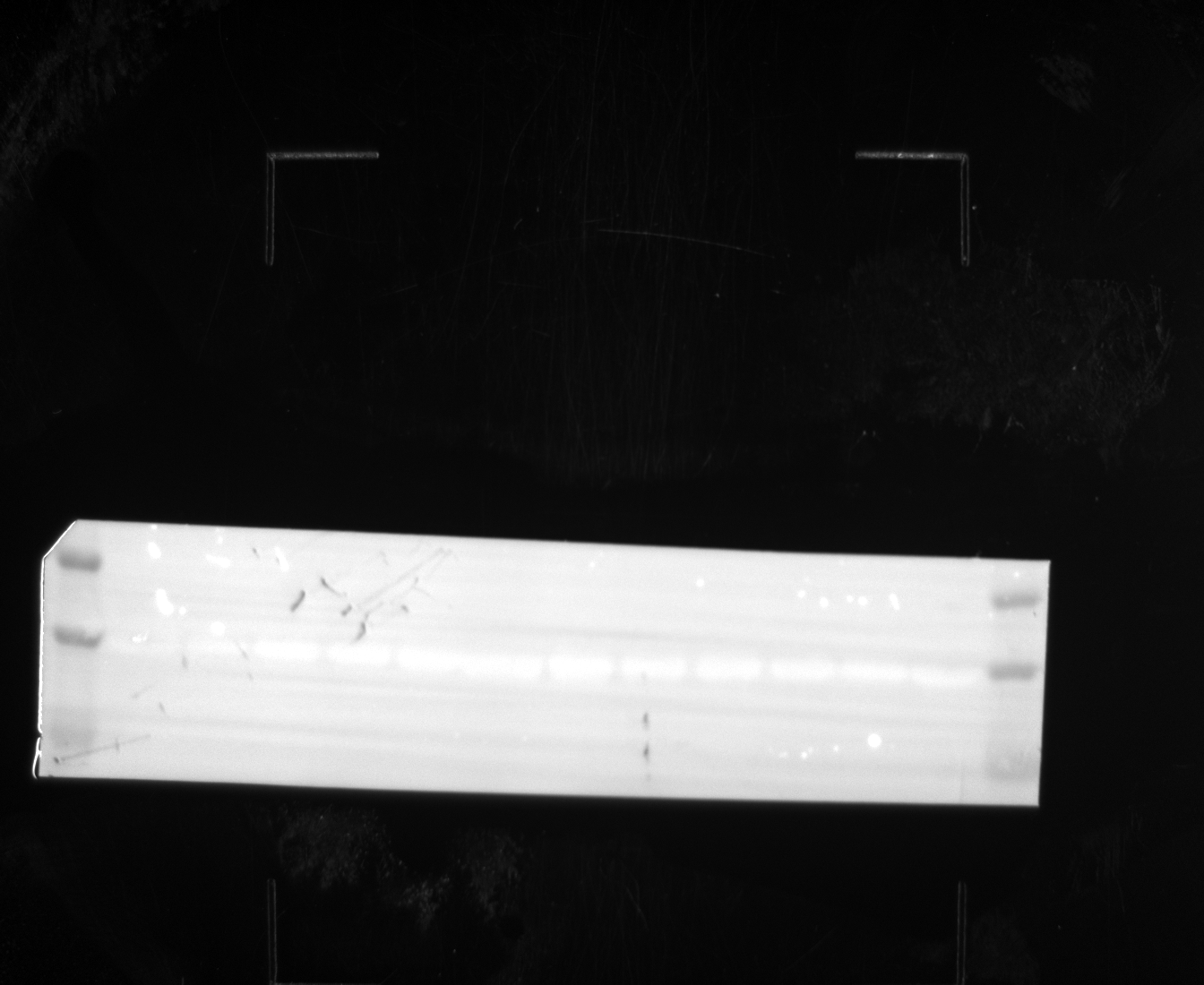

Supplement: Data S1. Raw unprocessed experimental imaging files sorted into three separate subfolders, covering Figure 1G GAPDH western blot raw images, Figure 2F 0 h scratch assay replicates of OSRC2 shNC cells, and Figure 3H 0 h scratch assay replicates of OSRC2 oeSBSN overexpression cells [file mmc2.zip › Raw data/Fig1G/GAPDH-tissue bright.tif]

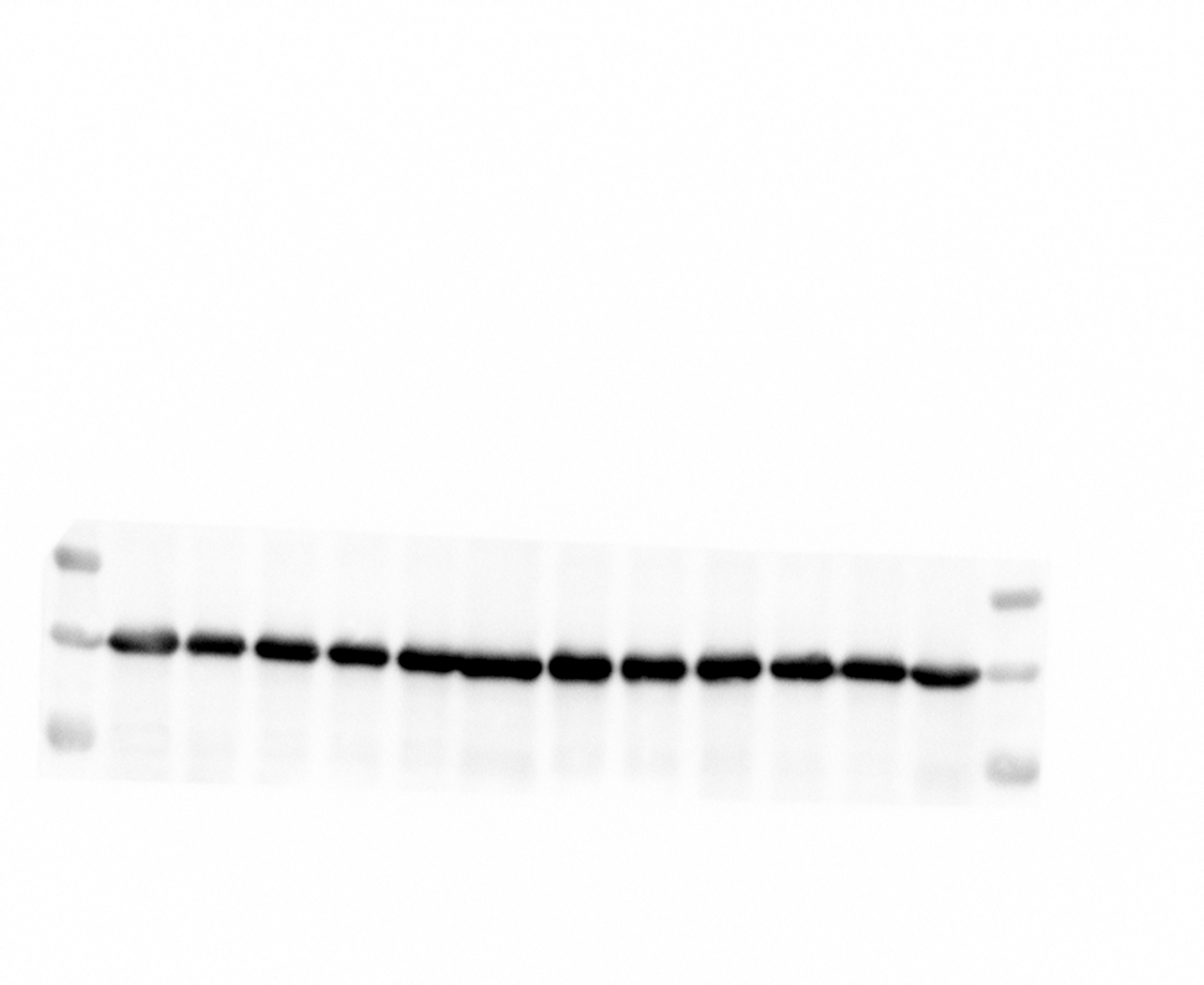

Supplement: Data S1. Raw unprocessed experimental imaging files sorted into three separate subfolders, covering Figure 1G GAPDH western blot raw images, Figure 2F 0 h scratch assay replicates of OSRC2 shNC cells, and Figure 3H 0 h scratch assay replicates of OSRC2 oeSBSN overexpression cells [file mmc2.zip › Raw data/Fig1G/GAPDH-tissue shine.tif]

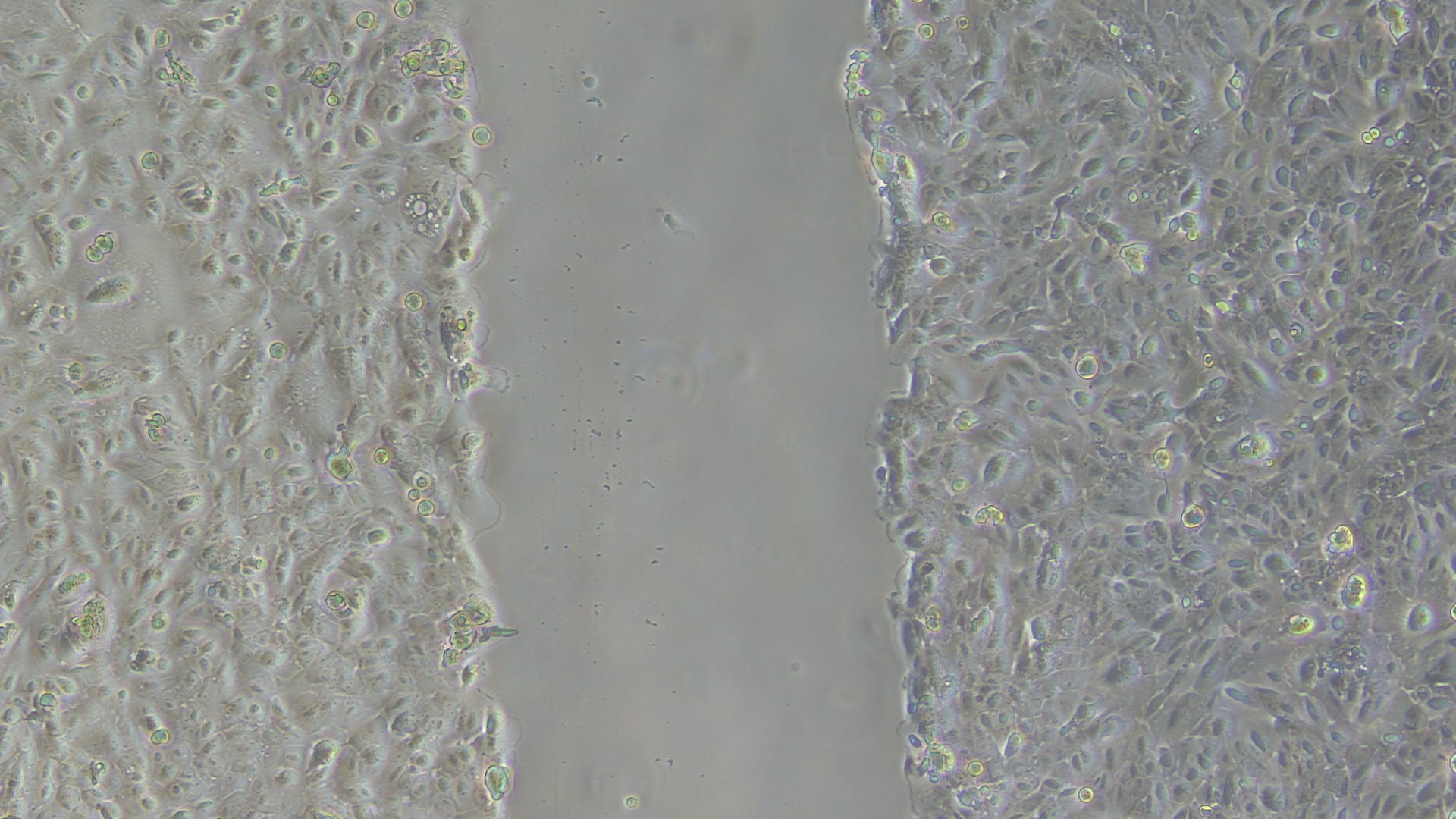

Supplement: Data S1. Raw unprocessed experimental imaging files sorted into three separate subfolders, covering Figure 1G GAPDH western blot raw images, Figure 2F 0 h scratch assay replicates of OSRC2 shNC cells, and Figure 3H 0 h scratch assay replicates of OSRC2 oeSBSN overexpression cells [file mmc2.zip › Raw data/Fig2F/IMG02738 OSRC2 shNC 0h rep1.JPG]

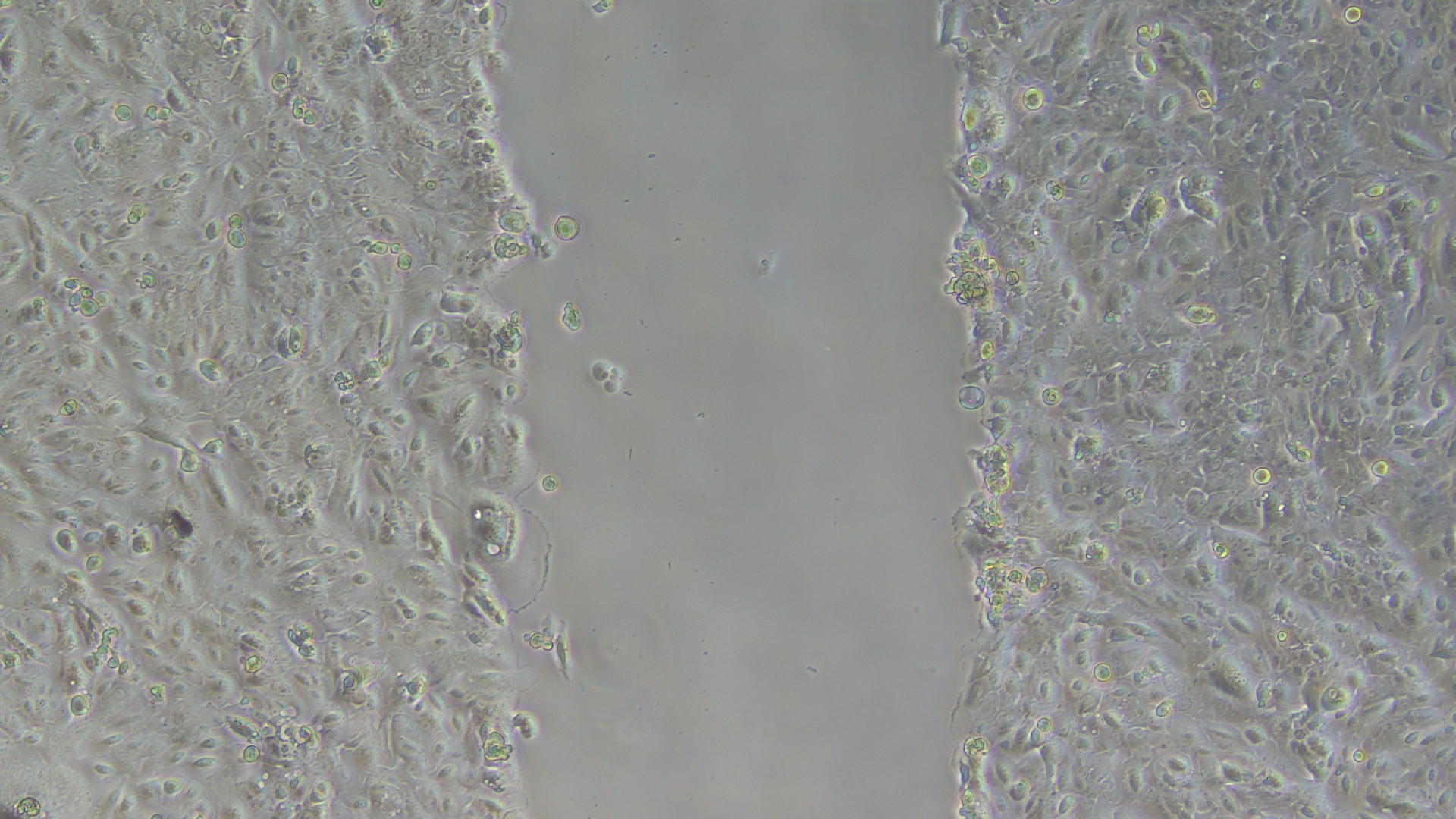

Supplement: Data S1. Raw unprocessed experimental imaging files sorted into three separate subfolders, covering Figure 1G GAPDH western blot raw images, Figure 2F 0 h scratch assay replicates of OSRC2 shNC cells, and Figure 3H 0 h scratch assay replicates of OSRC2 oeSBSN overexpression cells [file mmc2.zip › Raw data/Fig2F/IMG02739 OSRC2 shNC 0h.JPG]

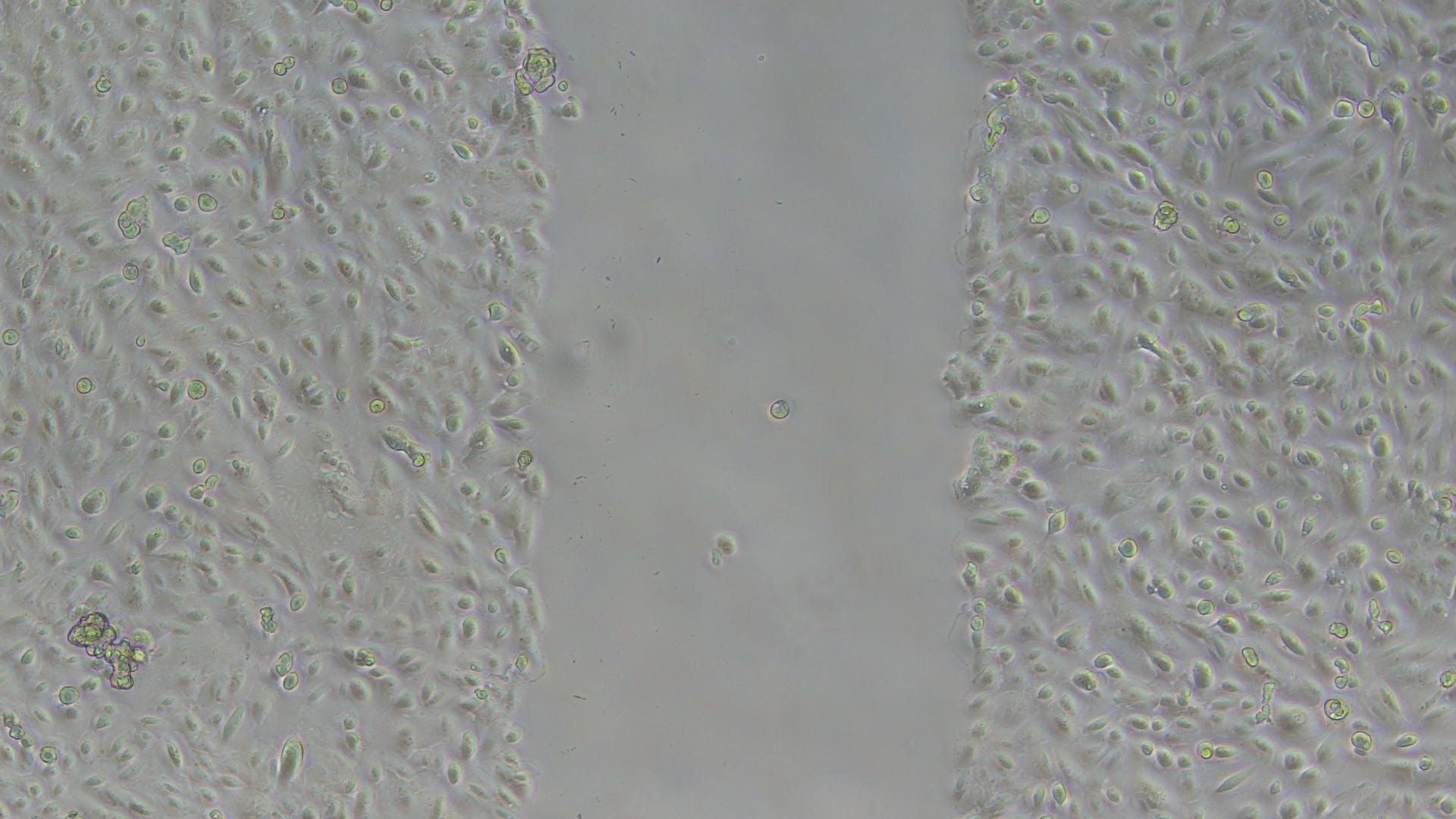

Supplement: Data S1. Raw unprocessed experimental imaging files sorted into three separate subfolders, covering Figure 1G GAPDH western blot raw images, Figure 2F 0 h scratch assay replicates of OSRC2 shNC cells, and Figure 3H 0 h scratch assay replicates of OSRC2 oeSBSN overexpression cells [file mmc2.zip › Raw data/Fig2F/IMG02740 OSRC2 shNC 0h rep2.JPG]

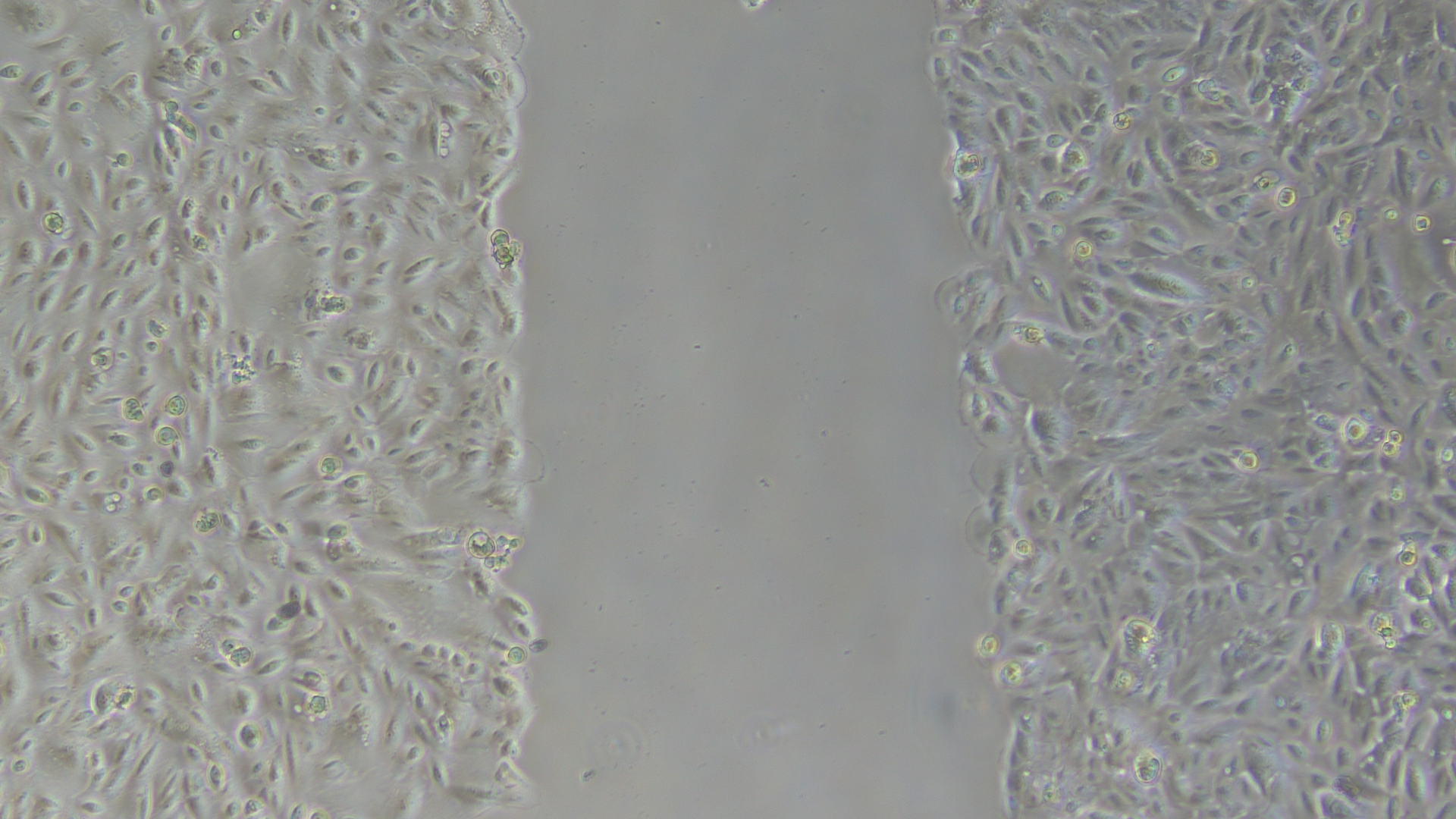

Supplement: Data S1. Raw unprocessed experimental imaging files sorted into three separate subfolders, covering Figure 1G GAPDH western blot raw images, Figure 2F 0 h scratch assay replicates of OSRC2 shNC cells, and Figure 3H 0 h scratch assay replicates of OSRC2 oeSBSN overexpression cells [file mmc2.zip › Raw data/Fig3H/IMG02709 OSRC2 oeSBSN 0h rep2.JPG]

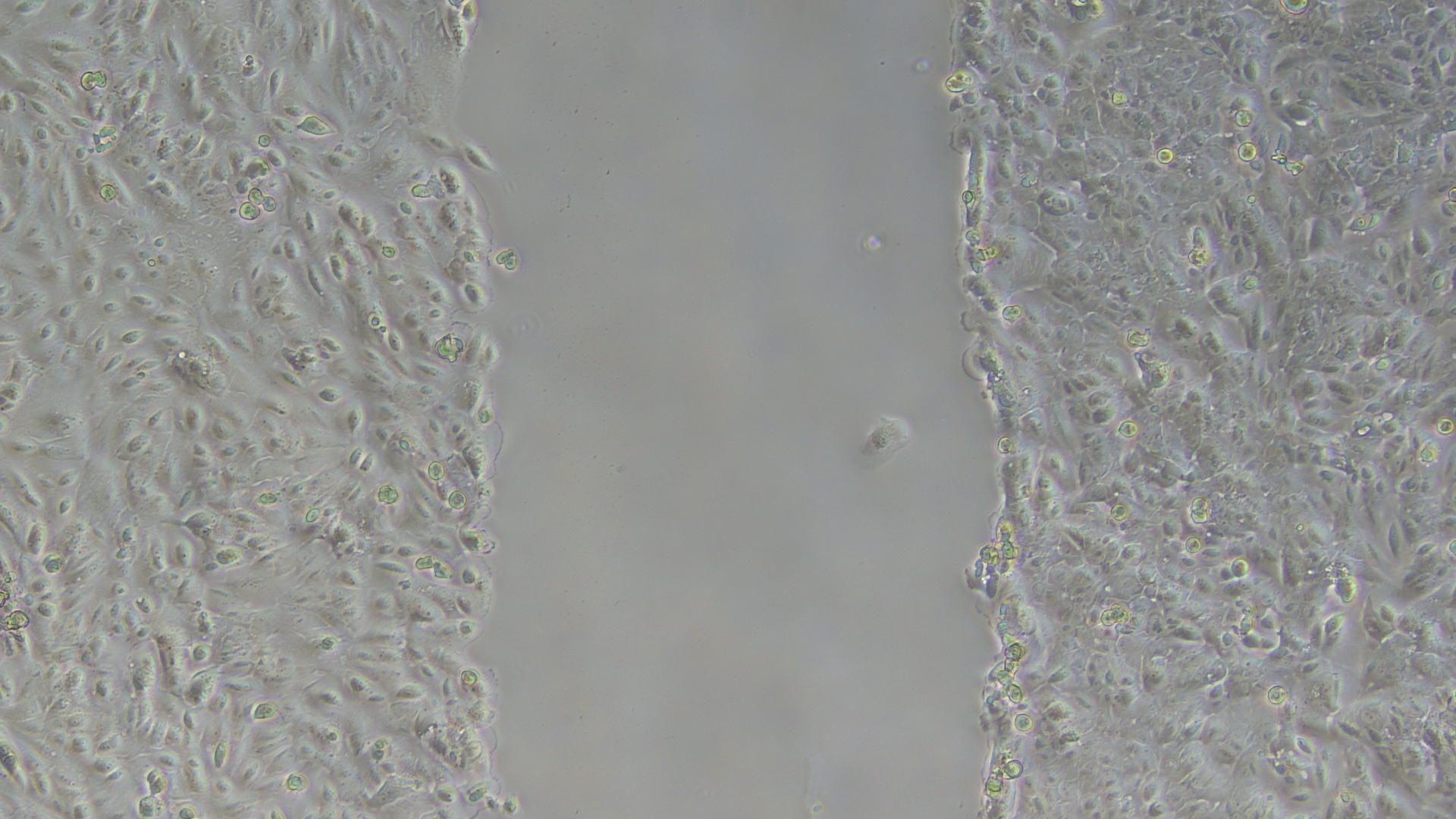

Supplement: Data S1. Raw unprocessed experimental imaging files sorted into three separate subfolders, covering Figure 1G GAPDH western blot raw images, Figure 2F 0 h scratch assay replicates of OSRC2 shNC cells, and Figure 3H 0 h scratch assay replicates of OSRC2 oeSBSN overexpression cells [file mmc2.zip › Raw data/Fig3H/IMG02710 OSRC2 oeSBSN 0h.JPG]

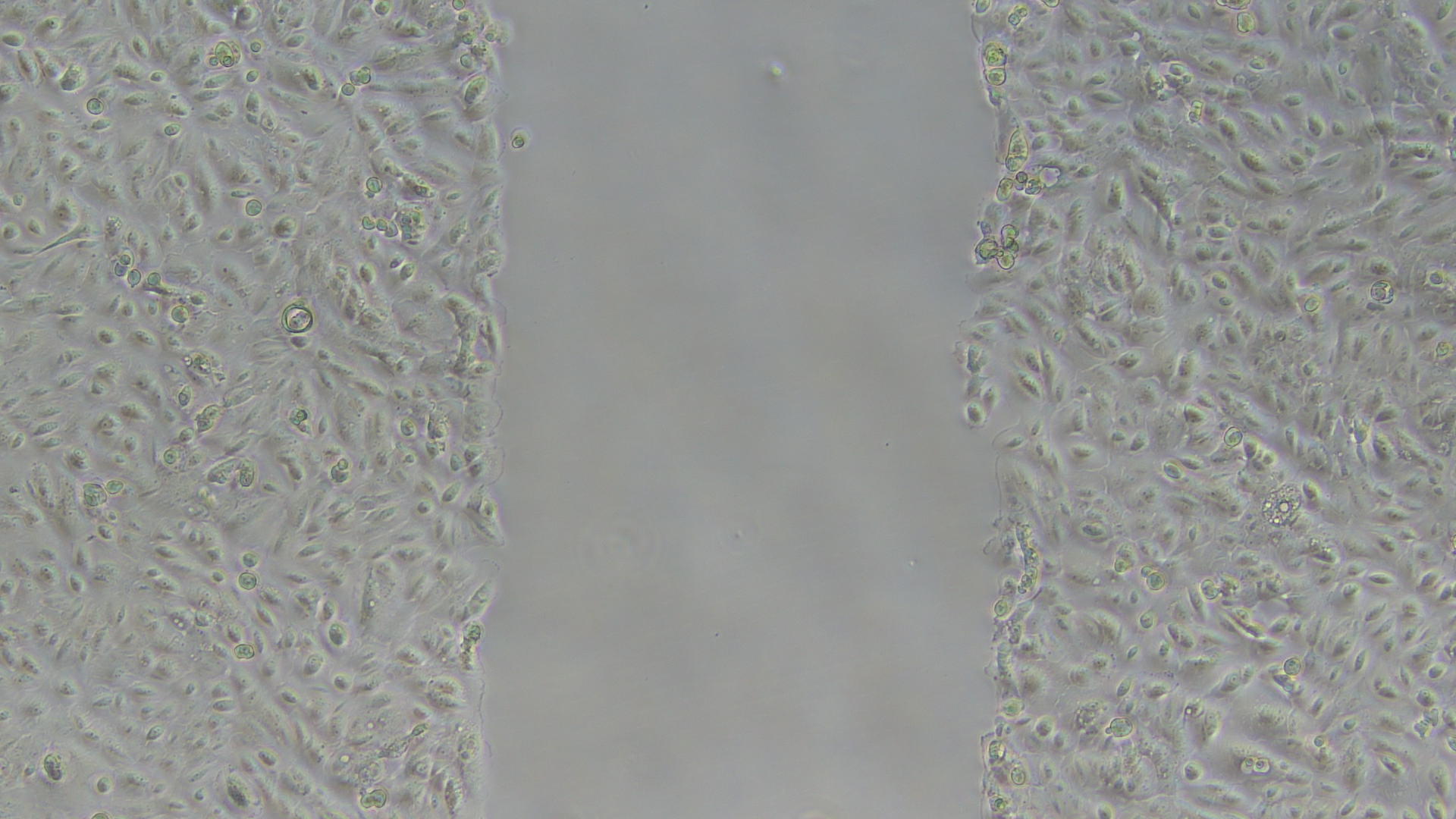

Supplement: Data S1. Raw unprocessed experimental imaging files sorted into three separate subfolders, covering Figure 1G GAPDH western blot raw images, Figure 2F 0 h scratch assay replicates of OSRC2 shNC cells, and Figure 3H 0 h scratch assay replicates of OSRC2 oeSBSN overexpression cells [file mmc2.zip › Raw data/Fig3H/IMG02711 OSRC2 oeSBSN 0h rep1.JPG]
